# The surface microstructure of cusps and leaflets in rabbit and mouse heart valves

**DOI:** 10.3762/bjnano.5.73

**Published:** 2014-05-13

**Authors:** Xia Ye, Bharat Bhushan, Ming Zhou, Weining Lei

**Affiliations:** 1 School of Mechanical Engineering, Jiangsu University of Technology, Changzhou Jiangsu 213001, China; 2 Nanoprobe Laboratory for Bio- & Nanotechnology and Biomimetics (NLB2), The Ohio State University, 201 W 19th Ave., Columbus, OH 43210, USA; 3 Center of Photonics Fabrication, Jiangsu University, Zhenjiang Jiangsu 212013, China

**Keywords:** contact angle, geometric parameter, heart valve, hemocompatibility, microstructure

## Abstract

In this investigation, scanning electron microscopy was used to characterize the microstructure on the surfaces of animal heart valve cusps/leaflets. The results showed that though these surfaces appear smooth to the naked eye, they are actually comprised of a double hierarchical structure consisting of a cobblestone-like microstructure and nano-cilia along with mastoids with a directional arrangement. Such nanostructures could play a very important role in the hemocompatibility characteristics of heart valves. On this basis, the model of the microstructure was constructed and theoretical analysis was used to obtain optimal geometric parameters for the rough surface of artificial valve cusps/leaflets. This model may help improve reconstructive techniques and it may be beneficial in the design and fabrication of valve substitutes or partial substitutes. Namely, the model may help ameliorate heart valve replacement surgery.

## Introduction

Bionics, or biomimetics, have made tremendous developments in the past decade due to advancements in nano- and biotechnologies. After millions of years of evolution and optimization, the surfaces of many organisms have formed a variety of special micro- and nanoscale hierarchical structures. These structures show many perfect characteristics such as superhydrophobicity, low adhesion, and drag reduction. Therefore, studying the surfaces of natural organisms is extremely important and significant. Moreover, results of this study could have a substantial effect on the manufacturing of artificial biological products. During the past decade, the special surface microstructures of plant leaves have been studied beginning with the lotus leaf [1–3]. Researchers then studied the microstructures of the India canna leaf, the rice leaf, and the leaf of *Colocasia esculenta* [4–5]. Subsequently, the study of surface microstructures expanded to animals. Researchers studied surface microstructures of the water skipper’s leg, the moth’s eye, shark skin, the darkling beetle, and the cicada’s wing [6–15]. At the same time, the relationship between superhydrophobicity and surface microstructures attracted strong interest. A large number of surfaces with all kinds of microstructures are manufactured by physical or chemical methods [16–22]. These manufactured surfaces have a wide range of applications in industry, agriculture, military and other areas [23–26].

Heart valves are located between the atria and both right and left ventricles, between the aorta and the left ventricle, and between the pulmonary artery and the right ventricle. These valves play a key role by forcing blood to flow in one direction through the heart and all blood vessels throughout the body. If valvular lesions occur a heart valve will need to be replaced. But once an artificial heart valve is implanted its surface changes by absorbing blood proteins, by platelet adhesion, and by the eventual formation of thrombi. Despite many efforts, blood clots are still an urgent and unsolved problem. In this paper, the surface microstructures of heart valve cusps/leaflets taken from animals are studied along with the effect of these microstructures on blood flow characteristics. Thus, this study will help improve the hemocompatibility of artificial heart valves.

## Experimental

**Sample preparation:** The thoracic cavities of the rabbits and the mice were cut open by using scissors and the hearts were removed. After irrigating with phosphate buffered saline (PBS), the hearts were fixed in a solution of 2.5% glutaraldehyde (Res Group Co., Ltd. chemical reagents) for 2 h. Then the atria and ventricles were cut open, and all of the heart valves (including aortic, mitral and tricuspid) were removed. The valves were placed in a freeze-dryer (ES-2030 vacuum freeze-drying device, Hitachi Japan) to be frozen, dehydrated, and dried with *tert*-butanol for 2–3 h at a temperature of −10 °C.

**Characterization of the microstructure on the surface of the heart valve:** The heart valves were observed by using the scanning electron microscope (S-3000N scanning electron microscope SEM, Hitachi Japan) at a voltage of 10 kV in order to characterize the microstructures on their surfaces. To improve the conductivity of the sample, before being observed the dried heart valves were treated with spray-gold (E-1010 ion sputtering device, Hitachi Japan) having a coating thickness of approximately 5 nm.

## Results and Discussion

### The microstructure on the surface of the aortic valve cusps

**Figure 1:**
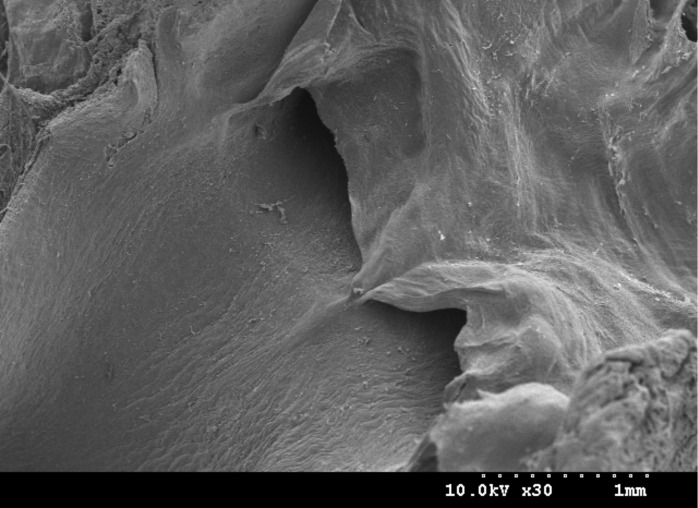
SEM image of the aortic valve of the mouse.

The aortic valve controls the direction of blood flow from the left ventricle to the aorta. When the ventricle is in systole, the intraventricular pressure increases dramatically. Until it exceeds the arterial pressure, the aortic valve opens. The blood from the left ventricle flows to the aorta and passes through the aortic valve. With the ending of ventricular pumping, the ventricle begins in diastole. Meanwhile, the intraventricular pressure rapidly decreases until it is equal to the arterial pressure, the aortic valve closes and blood does not flow backwards. The scanning electron microscopy (SEM) image of the aortic valve of the mouse is shown in Figure 1.

**Figure 2:**
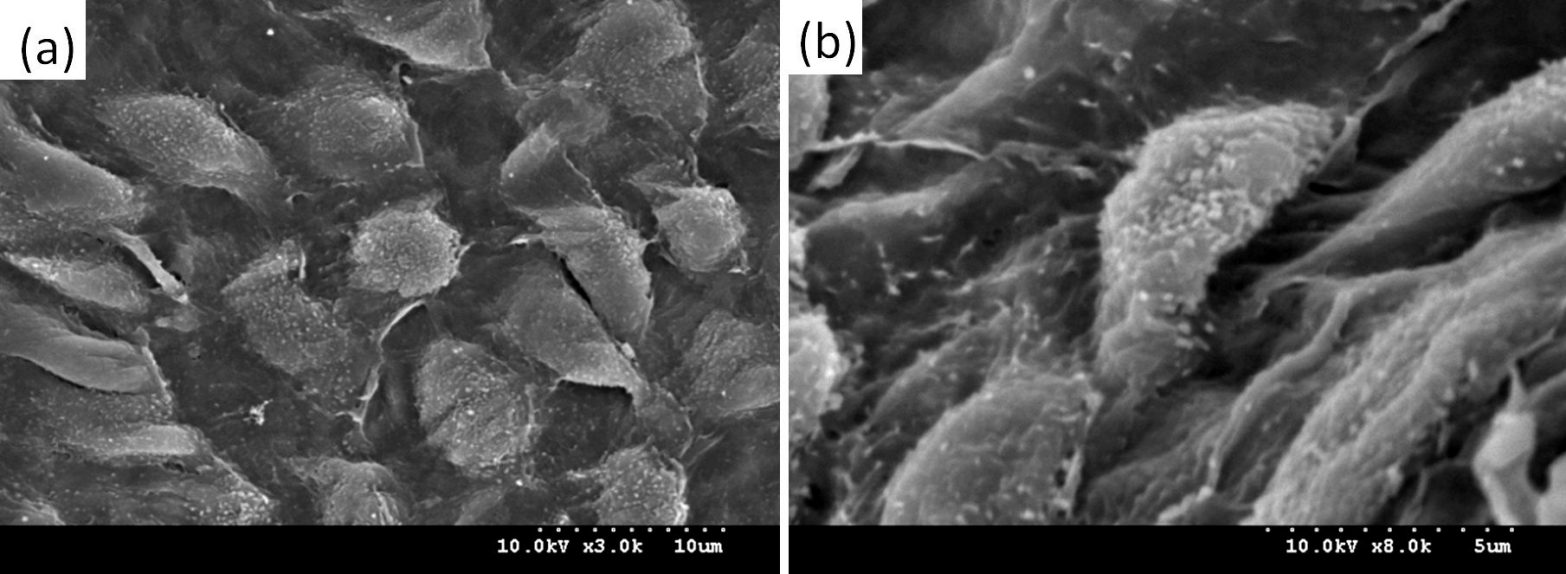
SEM images of the microstructure on the aortic valve cusps surface: (a) the cobblestone structure; (b) the cilia structure.

The microstructure on the surface of the aortic valve cusps is shown in Figure 2. It is evident from these images that a regular cobblestone-like structure appears on the surface of the heart valve cusps. This structure is similar to the microstructure on the surface of the lotus leaf. This cobblestone-like structure is uniformly distributed on the surface of the valve cusps and the bottom diameter of each “cobblestone” is approximately 5–9 μm. Figure 2b shows a high-resolution SEM image of the microstructure. In this image the hierarchical structure is formed of tenuous villi and “cobblestones”, and each villu has a diameter of 140–190 nm and a height of 350–500 nm. Because of the presence of a micro–nano composite hierarchical structure on the surface of the aortic valve cusps the actual contact area between blood and the surface of the valve is greatly reduced when blood flows through the valve. Thus the resistance to blood flow by the surface of the valve is reduced and the adhesion of blood platelets is also reduced. Eventually, the formation of blood clots is greatly reduced.

**Figure 3:**
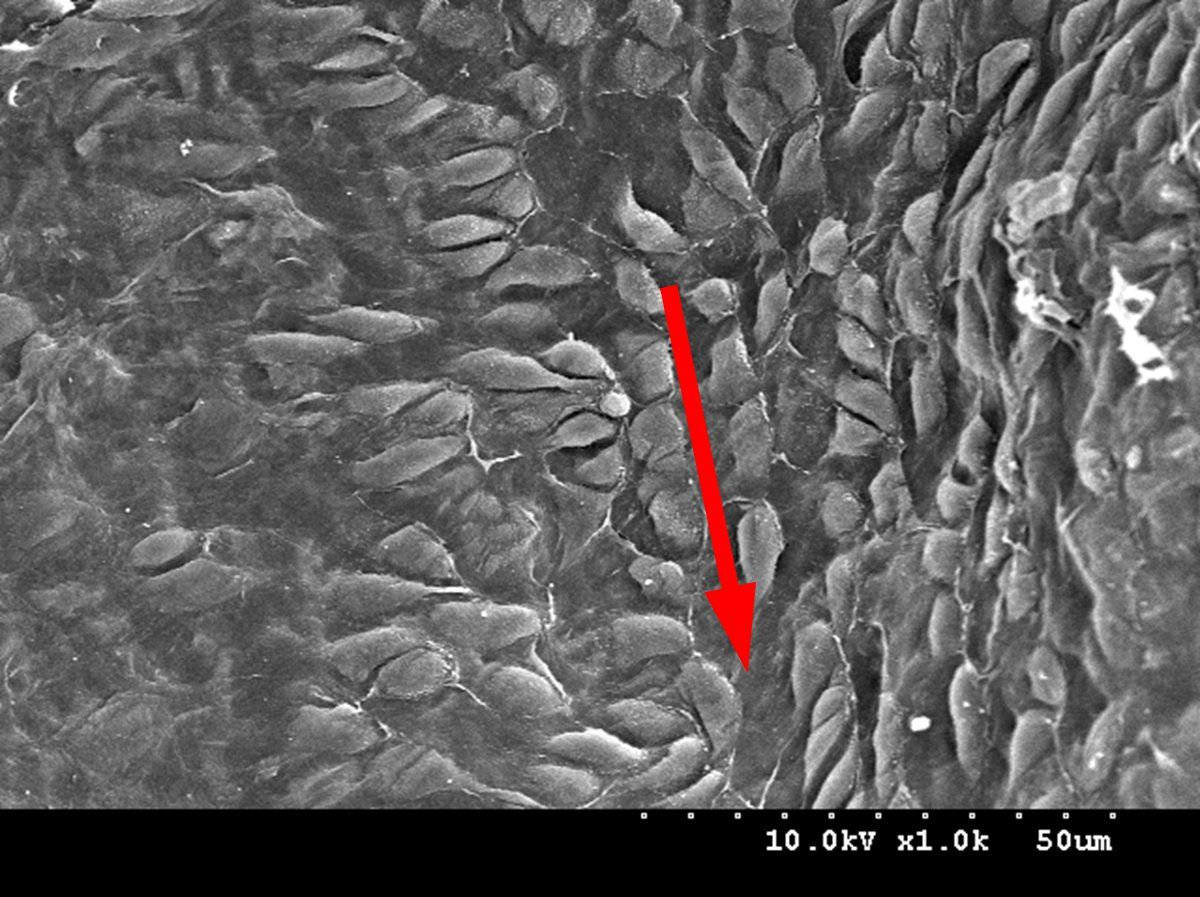
The direction of aligned cobblestones in the direction of blood flow.

Even more interesting, through the scanning electron microscope we found that the arrangement of the mastoids on the surface of the aortic valve cusps is directional. Figure 3 shows the arrangement of the mastoids along the blood flow of the aortic valve. It was found that the direction of the mastoids arrangement is the same as the direction of the blood flow, that is the same as the direction of the force transmission and it happens to be the same as the arrangement of the microstructure on the surface of the rice leaf studied by Jiang Lei [3]. Precisely because of the arrangement of this microstructure, blood flows more easily in the direction indicated by the red arrow than in the direction perpendicular to the arrow.

### The microstructure on the surface of the mitral valve leaflets

**Figure 4:**
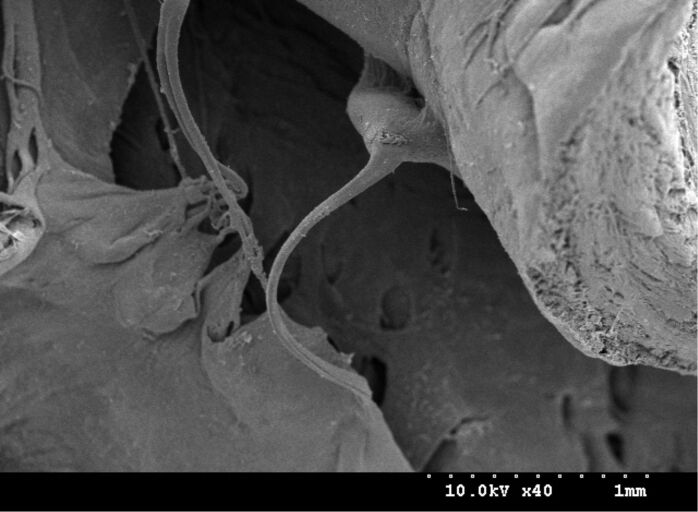
The mitral valve of the mouse.

Mitral is derived from the word “miter”, which is a bishop’s hat with two points. The mitral valve is used to control the unidirectional blood flow from the left atrium to the left ventricle. It is composed of two valve leaflets connected via the chordae tendineae to the papillary muscles, which are located in the myocardium (shown in Figure 4). When the atrium is in systole, the volume of the atrium decreases and then the atrial pressure increases, so the leaflets of the mitral valve open allowing blood to pass through the valve without difficulty to flow from the left atrium to the left ventricle. Subsequently, the atrium is in diastole and the ventricle is in systole. The papillary muscles located in the myocardium also contract with the contraction of the ventricle and the chordae tendineae are stretched. Then leaflets close to prevent the blood from backflowing. At this point, the leaflet is just like a sail and the chordae tendineae are just like ropes. Under normal circumstances, sails are filled by wind (that is the blood) and bellied out to their ideal bulge to control the direction of a ship (that is to ensure unidirectional blood flow). However, in the case of mitral valve disease, such as leaflets lengthy, weakness of myocardial contractility and ruptured or fused tendon causing uneven pulling force, some wind (blood) will pass through the sails (mitral valve leaflets) when the ventricle is in systole. This abnormal phenomenon may lead to the occurance of hemodynamic changes and the increase of cardiac load, causing cardiac hypertrophy, heart failure and other series of heart diseases.

**Figure 5:**
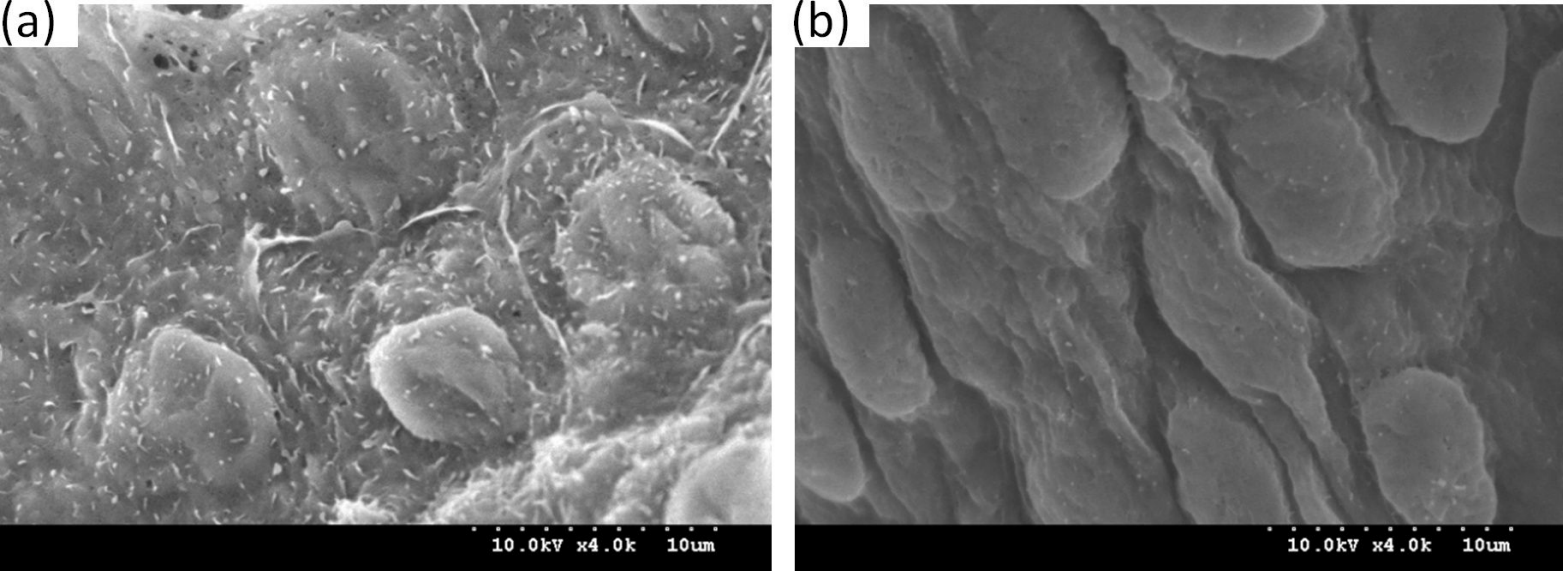
SEM images of the microstructure on the mitral valve leaflets surface: (a) non-heparinized; (b) heparinized.

The microstructure on the surface of the mouse’s mitral valve leaflets is shown in Figure 5. From this image, we find that the micro–nano composite hierarchical structure is also present on the surface of the mitral valve leaflets. Namely the distribution of the nano-scale cilia structure on the micron papillae is similar to the distribution of the microstructure on the surface of the aortic valve cusps. Without treatment by heparin there is a lot of mucus attached to the mitral valve leaflets surface. Therefore the mastoid structure is not obvious (shown in Figure 5a), but the cilia structure is seen clearly. The structures shown in Figure 5b were on the surface of the mitral valve leaflets that were treated with heparin. Because of rolling caused by heparin, the cilia structure was destroyed, so it is not obvious. However, because of the elimination of mucus, the mastoid structure became very clear.

**Figure 6:**
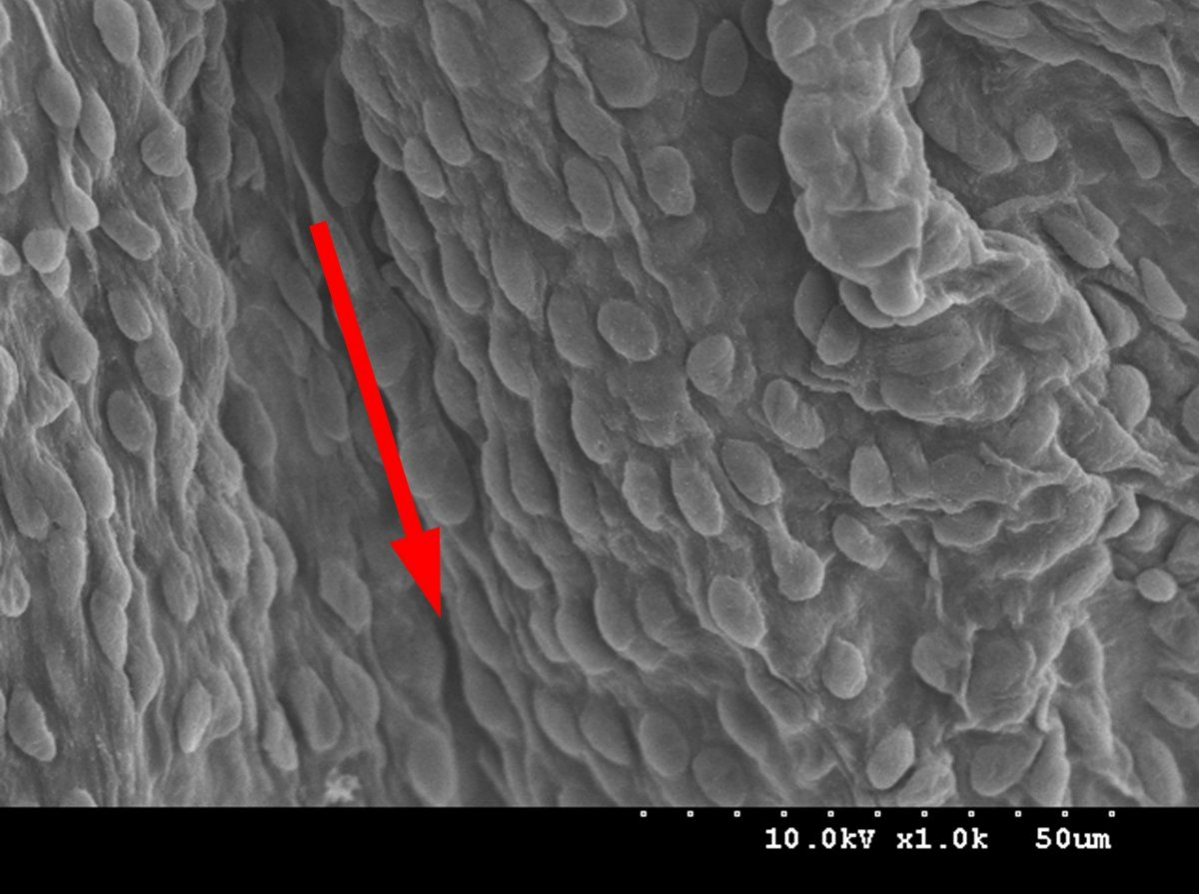
The direction of aligned “cobblestones” on the mitral valve leaflet’s surface.

In addition, the arrangement of the microstructure on the surface of the mitral valve leaflets can be seen as directional, and its direction is consistent with the direction of blood flow, as shown in Figure 6. The arrow in Figure 6 is pointed in the direction of blood flow. Compared to the blood flow in the perpendicular direction, the blood flowing in the direction of the arrow has less resistance and less adhesion of platelets to the valve leaflets’ surface will occur. It is mainly due to the proportion of the liquid–gas phase being larger during the liquid–solid–gas-phase contact between the valve’s surface and blood is larger, because of the increasing of the gap between the mastoids. Eventually, in the direction of the arrow, the formation of thrombus will be reduced.

### The microstructure on the surface of tricuspid valve leaflets

**Figure 7:**
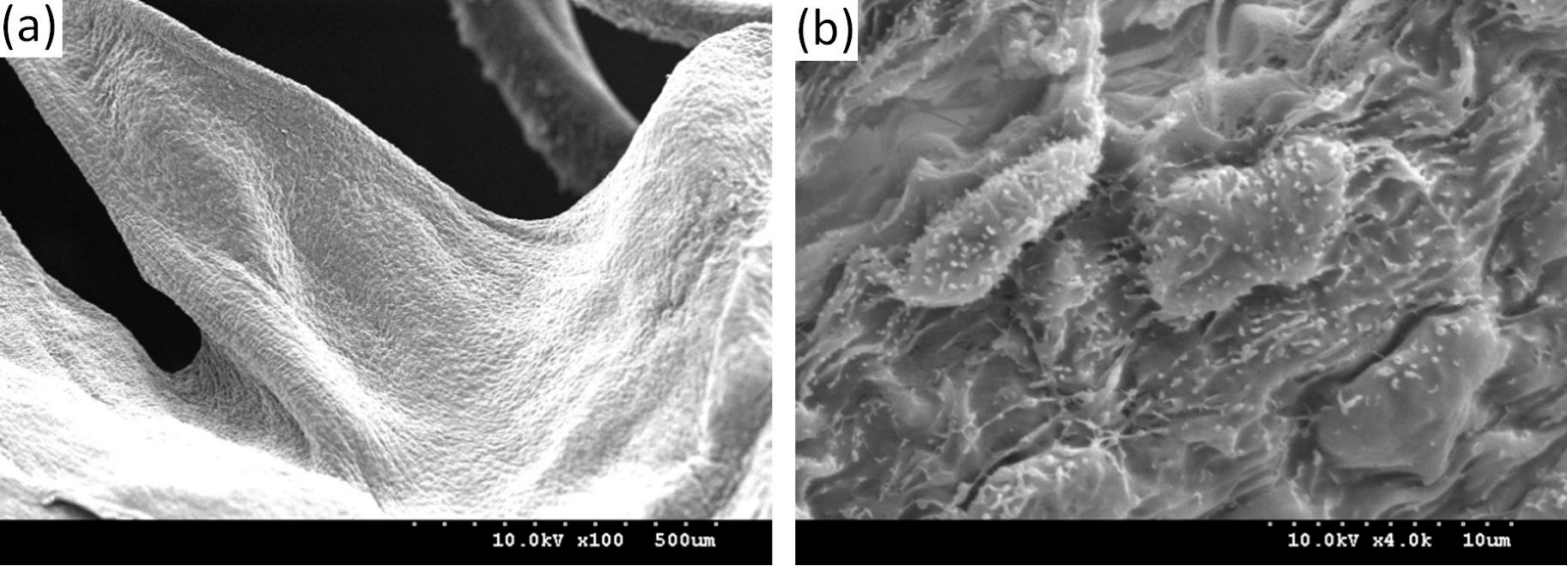
(a) SEM image of the tricuspid valve leaflets of the rabbit; (b) SEM image of the microstructure on the tricuspid valve leaflets surface.

The tricuspid valve controls the unidirectional flow of blood from the right atrium to right ventricle. Because it is composed of three leaflets it is called a tricuspid valve. The rabbit heart is 4 to 5 times larger than the heart of the mouse, so the tricuspid valve of the rabbit is larger than that of the mouse. Figure 7 shows the SEM images of the rabbit’s tricuspid valve leaflets. The structure of the tricuspid valve is similar to that of the mitral vlave. Through the chordae tendineae, the valve leaflets are also connected to the papillary muscles located in the myocardium, which is the same as the mitral valve. Additionally, both the mechanism and the time of opening and closing of these two kinds of valve are identical. When the atrium is in systole and the ventricle is in diastole, both of them open. On the contrary, when the ventricle is in systole and the atrium is in diastole, both of them close. The difference between them is that the tricuspid valve with three leaflets ensures the unidirectional flow of blood from right atrium to the right ventricle. The microstructure on the surface of the rabbit’s tricuspid valve leaflets is shown in Figure 7b. As shown in the figure, there are also micro- and nano-scale cilia mastoid composite hierarchical structures exsiting on the surface of the rabbit’s tricuspid valve leaflets.

Besides the aortic, mitral and tricuspid valves, there is another kind of heart valve, the pulmonary valve. The pulmonary valve controls the direction of blood flow only from the right ventricle to the pulmonary. The pulmonary valve often acts as an autologous substitute to replace the aortic valve during the Ross-operation, because they are very similar in structure and in the mechanism of openging and closing. However, the intraventricular pressure is different between the left and the right ventricle. Therefore, it is necessary to study the microstructure on the surface of the pulmonary valve cusps in order to find the differences and/or similarities of both valve cusps. Furthermore, according to the different directions of blood flow between atrial and ventricle, the orientation of the superficial structures on the surface of both sides of the mitral or tricuspid leaflets should be investigated. All of these will be our reseach interests in furture and this study will enormously help us to design and manufacture the microstructure on the surface of artificial vavle.

### Design and parameter characterization of the microstructure on the surfaces

Because of the hierarchical micro–nano composite structure, the surface of a heart valve is regarded as rough. According to Adamson and Gast [27], a model of the relationship between superhydrophobicity and the hierarchical structure could be created. Since the hierarchical structure on the surface of a valve is so similar to the fractal structure described by the Koch curve [28], the fractal structure equation can be used to calculate the fractal roughness factor. By changing the roughness factor, the relationship between the contact angle of the rough surface θ_f_ and that of the smooth surface θ_Y_ can be described by Equation 1:

[1]
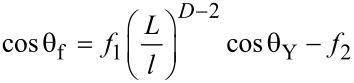


Where *f_1_* and *f_2_* are the surface area fractions of solid-liquid and gas-liquid on the rough surface respectively, *f*_1_ + *f*_2_ = 1. (*L*/*l*)^D−2^ is the surface roughness fractor. *L* and *l* are the extremal dimensions of the upper and lower limits of the fractal surface respectively. *D* is the fractal cone. For the heart valves, *L* and *l* correspond to the diameter of the cobblestones and the dimension of the nano-cilia respectively. In the Koch curve, *D* is about 2.2618 in three-dimensional space and (*L*/*l*) is 3*^n^,* where the size of *n* is determined by the specific fractal structure, and the surface roughness factor will increase with the increasing of *n*. Therefore, if the upper limit *L* is constant, the value of *l* will decrease with the increasing of the *n* value.

**Figure 8:**
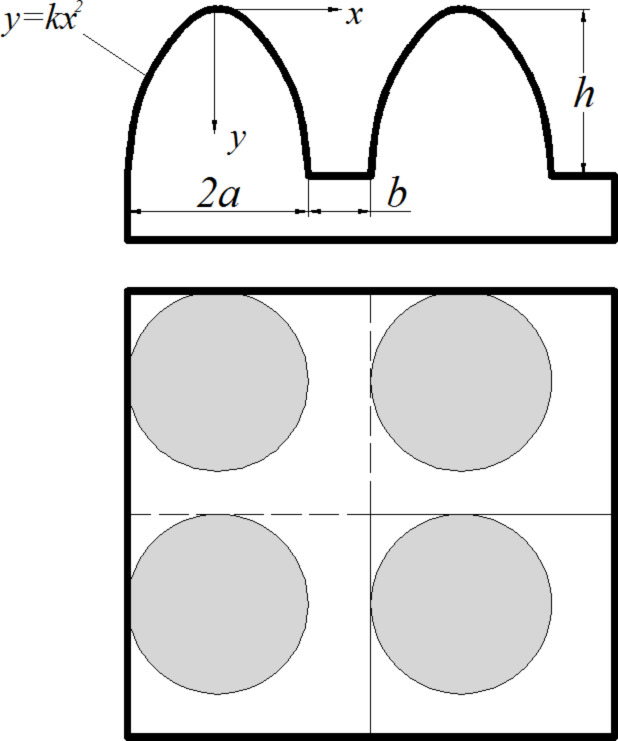
Sketch of the mastoid array microstructure: *a* is the basal diameter of a single mastoid, *b* is the space between two mastoids and *h* is the height of the mastoid.

In practical research, we always hope to construct more regular microstructures on surfaces in order to make research more convenient. According to the structure of the biological prototype, the familiar mastoid microstructure arranged in a regular array could be applied to the surface of an artificial heart valve. The front view and top view of the mastoid array microstructure is shown in Figure 8. The outline of the mastoid is assumed as paraboloid. The shape of a paraboloid of revolution is described by

[2]
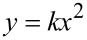


where *x* is the radial coordinate and *y* is the vertical coordinate measured downward from the apex (Figure 8). The higher the value of the constant parameter *k*, the steeper the shape of the paraboloid. The geometry size of a mastoid is as follow: basal radius *a*, spacing *b* and height *h*, as illustrated in Figure 8.

By investigating the one period lined out by the broken line frame in the top view of Figure 8, we will find that the actual area of the paraboloid is given by Equation 3. Therefore the total area of the rough surface is expressed by Equation 4.

[3]
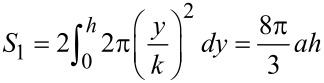


[4]
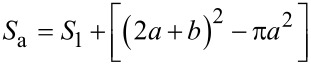


Its projected area is *S*_p_ = (2*a* + *b*)^2^, then, according to the definition, the roughness coefficient *r* will be given by the following Equation 5:

[5]
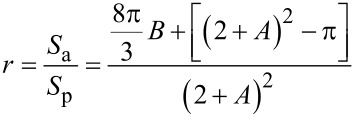


**Figure 9:**
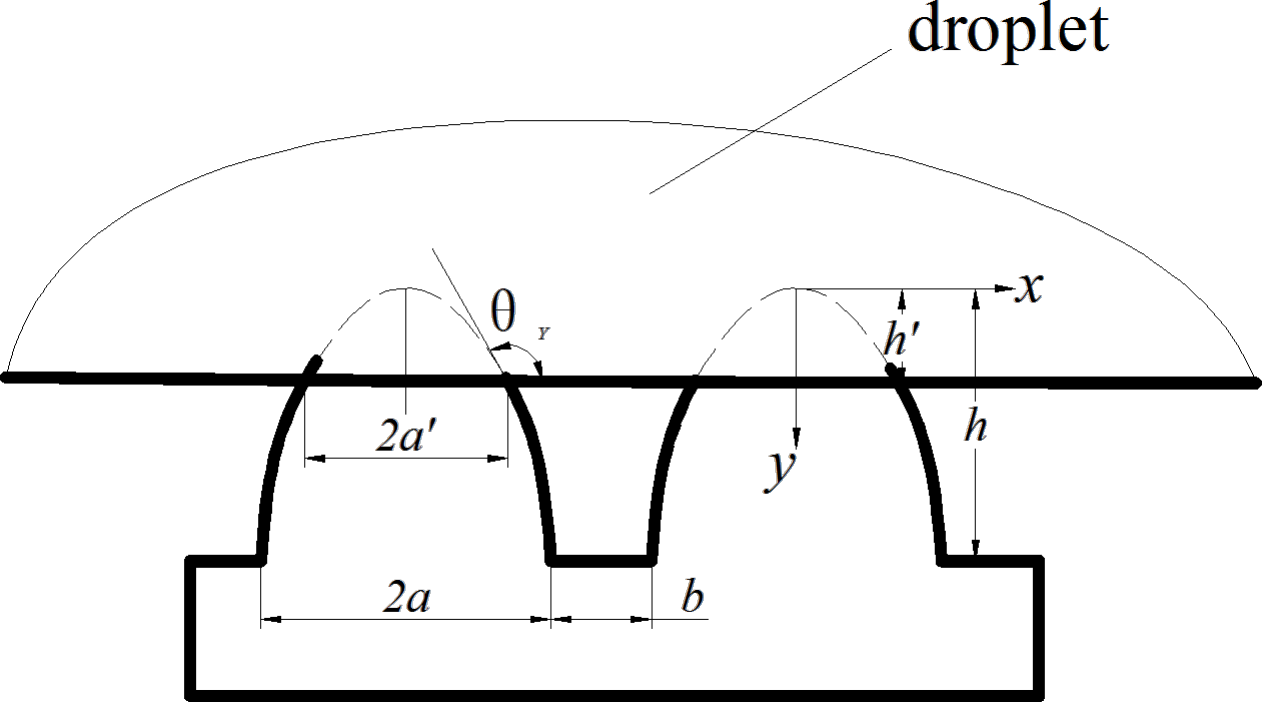
A droplet in Cassie state on the mastoid microstructure surface.

In this formula, two surface characteristic values, i.e., periodic space *A* (*A* = *b*/*a*) and aspect ratio *B* (*B* = *h*/*a*) are defined. When a droplet is at Cassie status, the bottom of it partially contacts with the top of the mastoid and the contact depth h′ is determined by the contact angle between the droplet and the solid (namely Young’s contact angle θ_Y_), which is shown in Figure 9. Since the size of the droplet is much larger than that of the microstructure, the bottom of the droplet can be approximated as a straight line and the contact area of the liquid–solid can be approximated as the small paraboloid with base radius a′, and height *h*′. According to Equation 3, the superficial area of the small paraboloid can be obtained as

[6]
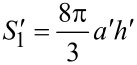


Since the equation of the parabola is *y* = *kx*^2^, at the interface, there is:

[7]
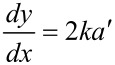


At the same time,

[8]
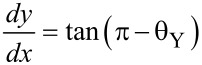


From Equation 7 and Equation 8, the base radius *a*′ can be given as

[9]
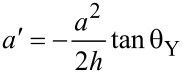


The height *h*′ can be obtained as

[10]
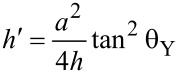


From Equation 9, Equation 10 and Equation 6 the area of the small paraboloid can be obtained as

[11]
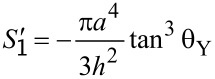


According to the definition, the area fraction *f* of the solid surface protuberance can be given as

[12]
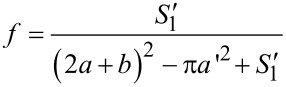


Therefore *f* can be obtained as

[13]
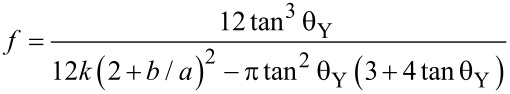


Substituting *r* in Wenzel’s equation [20–21] we will obtain the relationship between the apparent contact angle on the wetted surface, θ_W_, and Young’s contact angle θ_Y_ (i.e., the intrinsical contact angle).

[14]
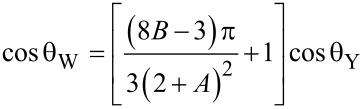


Substituting *f* in Wenzel’s equation [29–30] and Cassie’s equation [31–32], we will also obtain the relationship between the apparent contact angle on the composite surface θ_CB_ and Young’s contact angle θ_Y_.

[15]
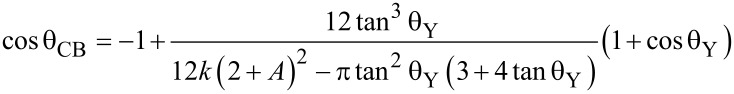


As can be seen from the above two equations, the apparent contact angle of Wenzel’s droplet on the mastoid microstructure arranged in a regular array is related to the periodic space, *A*, and the aspect ratio, *B*. The apparent contact angle increases with the increasing of *B* and decreases with the increasing of *A*. While the apparent contact angle of a droplet in Cassie state decreases with the increasing of *A*, but also relates to the steepness, *k*, of the mastoid structure.

Equation 14 and Equation 15 can be used as theoretically predicted formulas of the apparent contact angles on mastoid array microstructure surfaces for the two contact states of a droplet. They are also generally applicable equations for expressing the relationship between geometric parameters of mastoid array microstructures and the apparent contact angles.

## Conclusion

Learning from nature gives us the inspiration to generate topographic structures on artificial surfaces, which improves the characteristics of these surfaces. In this paper, we have studied the microstructures on the surfaces of a mouse and a rabbit’s heart valve cusps/leaflets by using SEM. The hierarchical structures can be found on the surfaces of heart valve leaflets from both the mouse and the rabbit. The hierarchical structure consists of a cobblestone-like microstructure and nano-cilia. The directional arrangement of the cobblestones-like microstructure and nanoscale cilia may greatly influence the properties of blood flow and anti-clotting. According to the morphology and microstructure of the biological surface, to simplify the design and manufacturing, the mastoid microstructures arranged in a regular array have been constructed on the artifical heart valve’s surface. Then theoretically predicted formulas of the apparent contact angles on such surfaces have been deduced from classical wetting theories. Next the proper geometric parameters of the microstructures that indirectly control the difference of the apparent contact angles should be decided. That will provide an intuitive guide for designing artificial heart valve surfaces. In later research, based on the above theorical study we should manufacture the mastoid microstructures on the biological materials surface. Further study of the relationship between anticoagulant properties and geometries of the microstructure through the experimental method is needed. Finding an artificial heart valve surface with better hemo-compatibility holds great significance for the replacement of mechanical heart valves.
